# Analysis of Excitability in Resonant Tunneling Diode-Photodetectors

**DOI:** 10.3390/nano11061590

**Published:** 2021-06-17

**Authors:** Weikang Zhang, Abdullah Al-Khalidi, José Figueiredo, Qusay Raghib Ali Al-Taai, Edward Wasige, Robert H. Hadfield

**Affiliations:** 1 Division of Electronic and Nanoscale Engineering, James Watt School of Engineering, University of Glasgow, Glasgow G12 8LT, UK; Abdullah.Al-Khalidi@glasgow.ac.uk (A.A.-K.); qusayraghibali.al-taai@glasgow.ac.uk (Q.R.A.A.-T.); edward.wasige@glasgow.ac.uk (E.W.); Robert.hadfield@glasgow.ac.uk (R.H.H.); 2 Centra-Ciências and Departamento de Física, Faculdade de Ciências, Universidade de Lisboa, Campo Grande, 1749-016 Lisboa, Portugal; jose.figueiredo@ciencias.ulisboa.pt

**Keywords:** resonant tunneling diode, photodetector, neuromorphic systems, stochastic dynamics, excitability

## Abstract

We investigate the dynamic behaviour of resonant tunneling diode-photodetectors (RTD-PDs) in which the excitability can be activated by either electrical noise or optical signals. In both cases, we find the characteristics of the stochastic spiking behavior are not only dependent on the biasing positions but also controlled by the intensity of the input perturbations. Additionally, we explore the ability of RTD-PDs to perform optical signal transmission and neuromorphic spike generation simultaneously. These versatile functions indicate the possibility of making use of RTD-PDs for innovative applications, such as optoelectronic neuromorphic circuits for spike-encoded signaling and data processing.

## 1. Introduction

Optoelectronic integrated circuits (OEICs) based on III-V semiconductor resonant tunnelling diodes (RTDs) achieve enhanced functionality in combination with optoelectronic devices, such as photodetectors [1,2,3], optical modulators [4], and lasers [5,6]. The negative differential resistance (NDR) of RTDs provides the intrinsic power gain, which can reduce the employment of extra complex transceiver building blocks [7,8] and makes the systems both compact and energy-efficient. In addition, RTDs are considered to be the fastest solid-state electronic devices at room temperature [9] thanks to the ultra-fast quantum mechanical resonance tunneling effect. To date, RTD-based devices have been developed and applied in several significant application scenarios, including high-bandwidth optical [10,11] and sub-THz wireless communications [12,13,14].

In recent years, research in photonic computing based on photonic integrated circuits has become a major topic in the domain of artificial intelligence [15]. RTD-based excitable optoelectronic devices have been proposed for emulating the excitability of biological neuron behaviors. The excitability generally describes the behavior in feedback systems (especially in biological neurons) that a weak external stimulus beyond a certain threshold can induce a strong response followed by a refractory period before returning to equilibrium [5,16]. During the refractory time, the device does not respond to stimuli. For RTD devices, the theoretical explanation of the excitability is elucidated in [17]. Excitable responses, such as spikes and bursts, act as the key mechanism of excitability in the dynamic behaviors of neurons and it is considered that information processing in the form of spikes is superior to traditional digital signaling and encoding [18]. In the past decade, an emerging field that combines both photonics and neuroscience has attracted much attention: Neuromorphic photonics can bring about important advances in ultra-fast neuro-inspired data processing [19,20], which is one of the next frontiers in computing [15,16]. The combined merits of high switching speed, high bandwidth, and low energy consumption in photonics and optics are suitable for ultrafast neuromorphic computing networks with high information density and high interconnectivity [21,22,23]. In this regard, RTD-based optoelectronic devices are considered candidates to meet the requirements of neuromorphic functionalities and future ultra-fast data processing technologies. Some studies and experiments on RTD-based optoelectronic devices have been demonstrated for stochastic spike generation or imitating certain neuron-like behaviors using controllable electrical or optical methods. For example, the dynamic behaviors of an RTD oscillator hybrid-integrated with a laser diode have been investigated in its monostable or bistable regimes [5,6] using electrical noise activation. Neuron-like logic-gate operations driven by electrical noise are demonstrated in [24]. Additionally, the light-induced stochastic dynamics of RTD-photodetectors (RTD-PDs) are illustrated in [25].

In this work, we experimentally study the dynamics and excitability of RTD-PDs, specifically focusing on the stochastic spike generation induced by either electrical noise or optical pulse signals. Several important operation conditions that directly control the output characteristics are investigated, including the operation point on the current-voltage (I-V) curve, the intensity of the input electrical noise and the optical signal. In the optical test, we also find that optical power with tens of photons per input pulse is adequate for inducing excitability. This makes such RTD-PD devices competitive candidates for adoption in energy-efficient optical neuromorphic networks. Besides, RTD-PD oscillators utilizing such RTD-PD devices have been implemented for optical data transmissions in previous work [11]. Thus, this work signposts the potential for applying RTD-PDs for stochastic spike generation and optical data transmission simultaneously. A series of measurements illustrate the versatile functions of RTD-PDs and provide a methodology for the implementation of RTD-based devices to obtain different output features.

## 2. RTD-PD Devices and Experimental Setup

**Figure 1:**
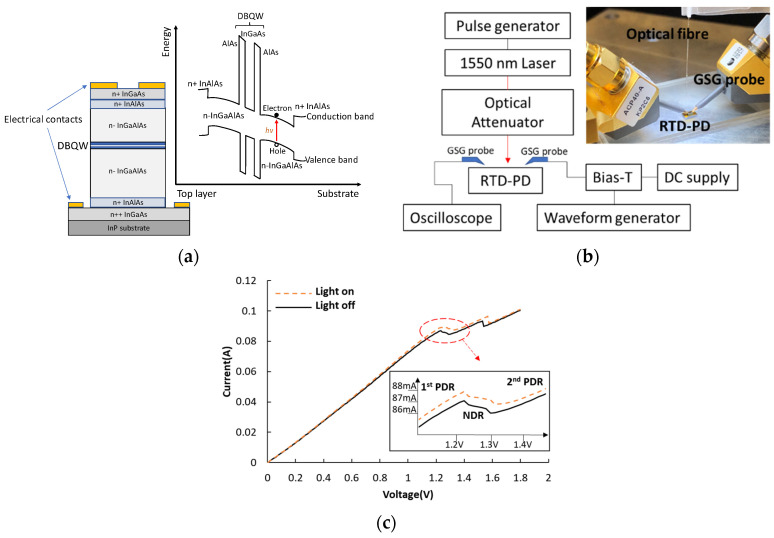
(**a**) Schematic diagram of the RTD-PD layer structure and the corresponding band structure, (**b**) schematic diagram of the experimental setup for electrical and optical-induced spike generation. The inset presents the RTD-PD under test with light injection, (**c**) I-V curves measured in light and dark conditions. The inset illustrates the N-shaped NDR region.

RTD-PD devices are essentially two-terminal semiconductor devices, consisting of vertical stacking of semiconductor epitaxy layers. The RTD-PD device under test was grown on an InP substrate with active layers comprising a narrow band-gap InGaAs layer (5.7 nm) sandwiched between two wide band-gap AlAs barrier layers (1.7 nm), forming a double barrier quantum well (DBQW) structure. The DBQW structure was then surrounded by two thick low-doped InGaAlAs spacer layers, which act as light absorption regions. A circular optical window with a diameter of 10 μm for light coupling is incorporated on the top mesa (10 μm × 10 μm). This InP/InGaAlAs system makes the RTD-PD devices feasible for optical detection at telecom wavelengths. Figure 1a shows the RTD-PD layer structure and the corresponding band structure. To realize an oscillator, passive components, including nichrome (NiCr) thin-film resistors, metal-insulator-metal (MIM) capacitors, and coplanar waveguides, are deposited in addition to the RTD device. Detailed design and fabrication procedures are set out in [26]. The typical NDR feature of RTDs is governed by the probability of electrons tunneling through the DBQW. The tunneling current increases with the bias due to a larger overlap between the Fermi sea of the incident electrons and the resonant energy levels within the quantum well, corresponding to the first positive differential resistance (PDR) region on the I-V characteristic. Beyond the peak current, further increasing the bias drops the transmission probability drastically, resulting in lower currents, as well as the NDR region. Higher applied voltage contributes to the thermal emission of electrons. This makes the observed current increase again after the valley current, forming the second PDR region [27]. There are two ports on the RTD-PD device: An input port for providing voltage and an output port for extracting the output response. For electrical noise excitation, an arbitrary waveform generator (AWG) was used for providing electrical noise, which was applied to the RTD-PD accompanied by a DC voltage through a bias-T (up to 4.2 GHz). For optical pulse excitation, only DC voltage was used for biasing and a pulsed light at a wavelength of 1550 nm was injected into the RTD-PD vertically via single-mode optical fiber. The optical intensity was controlled by an optical attenuator. For both tests, the output response from the device can be observed on an oscilloscope (Rohde&Schwarz RTO 1022, 2 GHz). The experimental setup is presented in Figure 1b. The RTD-PD is at room temperature in the ambient air, but internal heating may occur due to the current bias.

In this work, the RTD-PD is positively biased, meaning the top of the mesa is at a higher potential. The I-V characteristics in both light and dark conditions are illustrated in Figure 1c. The I-V curve shifts to the left and the current increases as a result of the light illumination [10]. Such an I-V curve shift towards lower voltages manifests a large part of the injected light is absorbed in the collector side of the device, leading to charge accumulation (particularly holes) adjacent to the collector-side barrier. Due to the existence of the resistance, which is designed as a stabilizing shunt resistor to suppress the low-frequency bias oscillations [2,28], the NDR region, as well as the light-induced I-V shift, is not pronounced. The RTD-PD operates in its self-oscillation state when the RTD is biased in the NDR region. When DC biasing in the first or second PDR regions slightly below the peak voltage or above the valley voltage, the RTD can respond to external perturbations if the intensity of the perturbation exceeds a given threshold, even though both biasing regions correspond to the steady-state of RTD. This study principally focuses on these two specific regions to investigate the excitability behaviors of RTD-PD in terms of external electrical and optical-induced perturbations.

## 3. Results and Discussion

**Figure 2:**
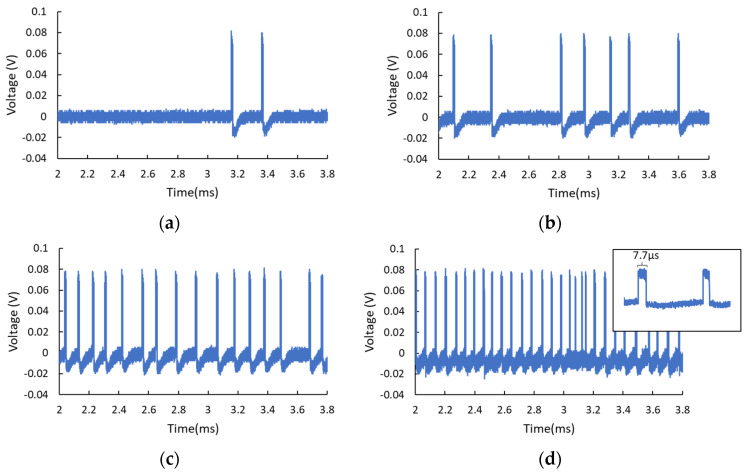

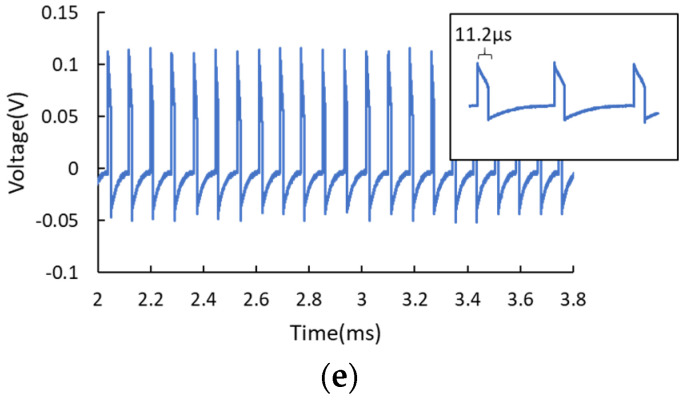
(**a**–**c**) The electrical-noise-induced stochastic spikes with increased noise intensity. (**a**) Noise amplitude = 50 mV, (**b**) noise amplitude = 56 mV, (**c**) noise amplitude = 62 mV, (**d**) for higher noise intensity of 90 mV, the output spikes tend to be periodic. The inset shows the spikes in the form of a square wave. (**e**) The self-oscillation when the RTD is biased in the NDR region without noise input. The inset illustrates the output waveform in this condition.

The RTD works in the steady-state when biasing at the end of the first PDR region. When applying electrical noise, the noise-driven perturbation may induce the emission of excitable spikes if the bias point is close enough to the NDR region. Figure 2 presents the temporal traces of the output waveforms observed on the oscilloscope. For relatively lower input noise intensity (50 mV), it is rare to observe excitable spikes, as shown in Figure 2a. With the increase of the noise intensity (56 mV), one can see the RTD emits more spikes, as shown in Figure 2b. It should be noted that, until this point, the noise-induced spikes fire randomly. Further increasing the noise intensity gradually drives the RTD into its self-oscillation mode, leading to a more stable regime of periodic pulse generation, as shown in Figure 2c,d, respectively, corresponding to noise intensity of 62 and 90 mV. This is the coherence resonance that was illustrated in [6]. As a comparison, Figure 2e presents the output of self-oscillation when completely biasing the RTD in the NDR region without electrical noise perturbation. One can notice the electrical-noise-induced pulses tend to be a square wave (inset of Figure 2d) and the amplitude is lower compared to the output pulses of self-oscillation (inset of Figure 2e). Additionally, the frequency of the output pulses for both situations can be tuned to some extent. For noise-induced spike generation, either higher applied voltage or higher noise intensity can result in more rapid spiking generation. It is also found higher noise density gives rise to shorter pulse durations. The pulse width presented in Figure 2a–d is around 10.5, 10.2, 9.9, and 7.7 μs, respectively. The voltage fluctuation of the pulses is up to 11 mV. In the case of self-oscillation, the oscillation frequency is 12.5 kHz with a pulse duration of 11.2 μs, as shown in Figure 2e, while higher applied voltages lead to higher frequencies due to the decreased equivalent capacitance. In this regime, the output pulses are stable with an amplitude variation of about 4 mV. These features are useful for designing RTD-based spiking generators in view of the operation speed, as well as the duration of the stimuli signal generated by the RTDs.

**Figure 3:**
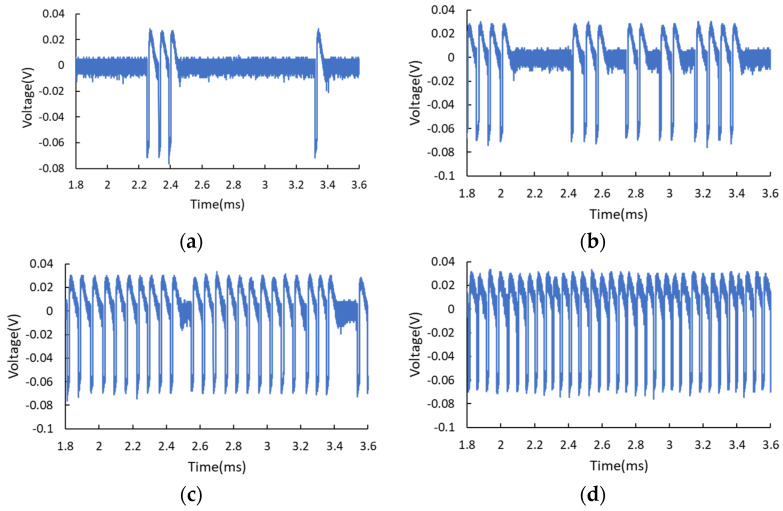
Temporal traces of the output waveforms when biasing the RTD at the start of the second PDR region. (**a**) Rare spikes for low noise intensity with noise amplitude = 30 mV. (**b**,**c**) Multi-pulsing bursts for moderate noise level, (**b**) noise amplitude = 34 mV, (**c**) noise amplitude = 38 mV. (**d**) Self-oscillation of RTD for higher noise intensities with noise amplitude = 60 mV.

Excitable spike generation can also be obtained by biasing the RTD at the start of the second PDR region, close to the end of the NDR region. Figure 3 illustrates the temporal traces of the output waveforms for this situation. Similarly, one can see more pulses are generated with the increase of noise intensity, as shown in Figure 3a–c, corresponding to noise amplitude of 30, 34, and 38 mV, respectively. However, the pulses excited are downward this time. Besides, biasing at the second PDR region results in multi-pulsing bursts and higher noise intensity gives rise to the increased clustering of the spikes. This phenomenon has been demonstrated and studied in [5,6]. The downward electrical pulses are considered due to the valley-to-peak transitions and the multi-pulsing bursting results from the asymmetric RTD I-V characteristic. Once again, the RTD operates in the oscillation mode (or coherence resonance) for higher noise intensity of 60 mV, with output amplitude fluctuation of about 14 mV, as shown in Figure 3d. For both biasing situations (i.e., biasing at the end of the first PDR or the start of the second PDR), the number of spikes can be partially controlled by simply adjusting the applied voltage or the intensity of the electrical noise. The closer the biasing point approaches to the NDR region, the less noise intensity is required to obtain similar excitations due to a lower excitable threshold needed.

**Figure 4:**
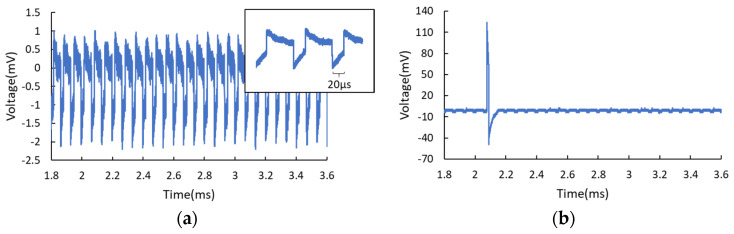

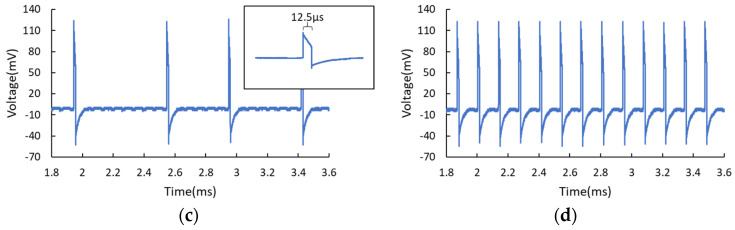
(**a**) For RTD biased in the 1st PDR region far from the NDR region (V = 1.1490 V), it responds to the modulated light and generates periodic square waves. The average optical power was kept constant at 87.6 μW. The inset in (**a**) presents the output of the RTD-PD showing 20 μs downward square waves corresponding to the 15 kHz, 30% duty cycle modulated light signal. With the gradual increase of applied voltage, more light-induced stochastic spikes are generated, as illustrated in (**b**) V = 1.1524 V, (**c**) V = 1.1525 V, (**d**) V = 1.1530 V. The inset in (**c**) indicates the spike whose width is independent of the frequency of the light. Noticeably, the periodic square waves can coexist with stochastic spikes.

The stochastic spikes generation can also be activated by light due to the existence of InGaAlAs spacer layers. The optical absorption coefficient of these optical absorption layers covers the telecom wavelength range of 1310–1550 nm. The frequency of the light pulse train was set as 15 kHz, which is close to the self-oscillation frequency of RTD. The duty cycle was set as 30%. When biasing the RTD at the first PDR region, far from the NDR region (e.g., V = 1.1490 V), the RTD-PD works in the steady-state and responds to the modulated light, as shown in Figure 4a. The inset illustrates the output response of the RTD, showing the downward square waves with 20 μs pulse width, conforming to the 15 kHz, 30% duty cycle light signal. The average optical power was kept constant at 87.6 μW, and the applied voltage was gradually increased, approaching the NDR region. One can observe the emergence of stochastic light-induced spikes, and the number of spikes rises with the increase of DC bias, as presented in Figure 4b–d, corresponding to the applied voltage of 1.1524, 1.1525, and 1.1530 V, respectively. When the applied voltage approached 1.1530 V, the emission of spikes tended to be more periodic. One can see the light-induced spikes can coexist with the square pulses responding to the modulated optical signal. The width of the spikes is 12.5 μs, which is dependent on the applied voltage and the intrinsic property of the oscillation circuit, instead of the modulated light signal. We also conducted a test that keeps the applied voltage constant and only adjusts the optical intensity. For low optical intensity, light-induced spiking pulses are generated occasionally. With the increase of optical power, more spikes are generated. Eventually, the RTD works steadily in the oscillation mode for higher optical power due to the light-induced I-V shift. Less optical intensity is required for the excitation if one biases the RTD closer to the NDR region. Increasing the optical power brings about the same effect as increasing the electrical noise level, as demonstrated in the foregoing measurement. However, the optically induced spikes do not possess obvious amplitude fluctuations compared to that of electrical noise-induced pulses. In addition, the interval between pulses can be adjusted by tuning the optical intensity because the internal capacitance of RTD is directly affected by light through the carrier accumulation effect. The oscillation frequency can be tuned by several kilohertz with optical power variation of tens of microwatts, depending on different optical window sizes. This further enhances the dynamics and offers an alternative way to control the excitable wave response. In addition, changing the frequency of the optical signal scarcely affects neither the width nor the temporal randomness of the spikes.

**Figure 5:**
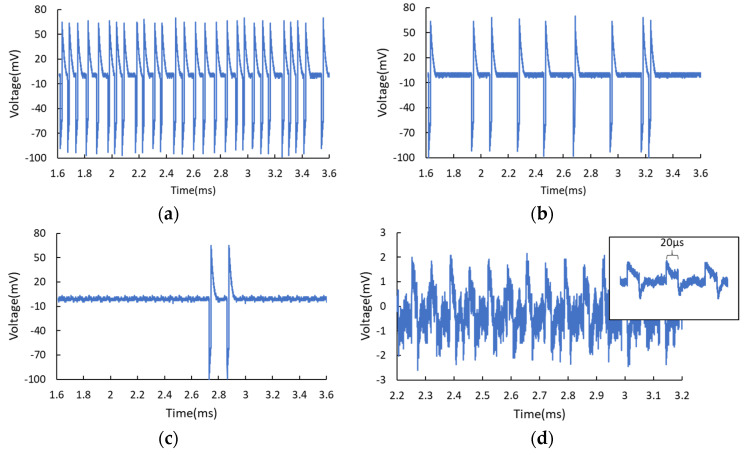
(**a–c**) The time traces of the RTD-PD output in terms of different optical intensities when the RTD was biased at the end of the NDR region. Without light, the RTD almost works in the self-oscillation mode, as shown in (**a**). (**b**) For optical power of 2.69 μW, spikes tend to be non-consecutive, random, and spaced out in time. (**c**) For higher optical power of 87.6 μW, fewer spikes are generated randomly. (**d**) The response corresponding to the modulated light when the RTD operates in the second PDR completely. The inset indicates the upward pulses with a pulse width of 20 μs.

However, when biasing at the interface between the NDR and the second PDR region, increasing the optical power may bring about contrary results. In this measurement, no light was injected initially, and the bias point was chosen at the end of the NDR region close to the start of the second PDR region, meaning that the RTD operates in the self-oscillation mode. In this situation, weak perturbations may interfere with stable self-oscillation and bring about random breaks. The amplitude fluctuation is higher (up to 12.6 mV) because of the thermal emission of electrons when biasing at this region, as shown in Figure 5a. With the increase of optical power, periodic oscillation tends to be across consecutive pulses, and higher optical power makes the RTD emit fewer spikes, as shown in Figure 5b,c, corresponding to the optical power of 2.69 and 87.6 μW. In Figure 5c, one can see weak pulses induced by the modulated light. Eventually, spikes disappear completely, and the RTD may operate in its steady-state because the operating point is now in a new second PDR region due to light-induced I-V shift. As a comparison, Figure 5d shows the response of RTD-PD to the modulated light signal when further biased completely in the second PDR region. One can see the pulses are upward with a pulse width of 20 μs. When biasing in the second PDR region, a large amount of the current is contributed by the thermal emission of electrons [26,27], thus bringing about more thermal noise to the output signals.

In this work, we also investigate the minimum optical intensity that can drive RTD-PD for stochastic spike generation at room temperature. By attenuating the optical intensity, optical power of 0.08 nW, corresponding to around 31 photons per pulse for the 1550 nm pulsed light at 2 MHz, on the rough assumption that 10% of the light is absorbed by the device, can induce an observable photocurrent and weak spiking as long as the bias point is close to the NDR region. The absorption efficiency is evaluated based on the usage of single-mode fiber (SMF-28) at an operating wavelength of 1550 nm with a mode field diameter of 8.2 μm and a numerical aperture of 0.14 [29]. The estimated separation between fiber tip and detector is controlled between 500 and 800 μm. This result implies that the optical intensity required for neuromorphic spiking is extremely low. Implementation with still lower photon number per pulse may potentially be achieved by reducing the operating temperature and improving the optical absorption efficiency, e.g., using increasing the window size and introducing anti-reflection coating. On the other hand, the total energy consumption of the RTD-PD can be reduced by designing nano-RTD devices with μA current level, which is beneficial for an artificial neural network with a high interconnection density.

## 4. Conclusions

We have investigated the excitability of an RTD-PD that can be activated by both electrical noise and modulated optical signal. The characterization of the excitability can be summarized as below: The electrical noise, as well as the injected light, plays the role of external perturbations that drive the RTD to perform the stochastic spike generation. The closer the biasing point approaches the NDR region, the lower the intensity of the perturbation required to achieve the same excitable behavior. For electrical noise activation, one can observe three different behaviors depending on the biasing positions. When biasing at the end of the first PDR region, moderate noise intensity leads to stochastic spike generation and high noise intensity leads to the so-called coherence resonance. While biasing at the start of the second PDR region, the multi-pulsing bursting can be obtained and the clustering of the spikes can be controlled by the noise intensity. As for the optical signal-induced excitability, the random spikes can coexist with the regular responses caused by the modulated optical signals. The versatility of RTD-PDs in performing multiple functions by simply controlling a few operating parameters brings about the potential that RTD-PDs can function with different neuromorphic spiking behaviors and optical signal transmission without additional electronic or optoelectronic elements. Besides, optically induced spiking on RTD-PD can be achieved with quite low optical intensity at the level of tens of photons at room temperature. Improved performance can be targeted through further optimization of the absorption efficiency and experimental conditions. The thermal issue within the device that results in undesirable current drift and thermal noise will be handled by improved heat regulation. A low-temperature test using cryostat can be conducted to mitigate the influence of internal and ambient heat and to further investigate the feasibility of photodetection with a lower photon number per optical pulse. The design of nano-RTD-PDs with reduced areas in future work can also reduce energy consumption, which is a major advantage of photonic artificial neuromorphic networks. In summary, this work demonstrates the feasibility of applying RTD-PD-based optoelectronic circuits for innovative neuromorphic applications, data processing, and transmission. 

## Data Availability

Publicly available datasets were analyzed in this study. This data can be found at https://www.gla.ac.uk/research/enlighten/ (accessed on 6 May 2021).
